# A gathering of minds: expanding understanding of the origins of biological diversity and the evolution of developmental mechanisms

**DOI:** 10.1186/2041-9139-3-5

**Published:** 2012-02-23

**Authors:** Christine A Byrum

**Affiliations:** 1 College of Charleston, Rita Liddy Hollings Science Center, 58 Coming St., Room 214, Charleston, SC 29401, USA

**Keywords:** development, evolution, larval, lophotrochozoa, deuterostome, vertebrate, cnidarian, ctenophore, nematode, meeting

## Abstract

This paper is a short report on the 2012 Society of Integrative and Comparative Biology Annual Meeting. Charleston, South Carolina, USA. 3-7 January 2012 (abstracts freely available at http://www.sicb.org/meetings/2012/).

## Main text

This year, a record number attended the Annual Society of Integrative and Comparative Biology (3-7 January 2012) meeting in Charleston, South Carolina. The meeting offered a wide range of talks about the evolution of development, as well as sessions in the other SICB divisions (animal behavior, comparative biomechanics, comparative endocrinology, comparative physiology/biochemistry, ecology/evolution, invertebrate zoology, neurobiology, phylogenetics/comparative biology and vertebrate morphology). This report summarizes a few of the many excellent presentations (800+ talks/600+ posters), focusing on those in the evolution of development.

## Looking at the lower metazoans

Among the lower metazoans, understanding of the ctenophores is clearly progressing. William Browne in collaboration with Christine Schnitzler, Derek Gildea, Anh-Dao Nguyen, E. Maxwell, Joseph Ryan and Andreas Baxevanis (University of Miami, USA; NHGRI/NIH, USA) presented preliminary analysis of next-generation RNAseq data from *Mnemiopsis leidyi*. In this study, they examined mRNA and smRNA expression at two developmental stages, zygotes 0 to 1 hours post fertilization (hpf) and late embryonic stages (15 to 30 hpf). Interestingly, they found elevated levels of two *Kruppel-like factor (Klf) *genes in zygotes. In light of J. Jiang's recent work showing that *Klf *is required for self-renewal of mouse embryonic stem (ES) cells and that ES cells depleted of *Klf2*, *Klf4 *and *Klf5 *undergo differentiation, Browne *et al*. suspect that ctenophore maternal *Klf *transcripts may aid in regulating pluripotency. They have also found a complex zygotic transcriptome, suggesting that maternal transcripts strongly influence early cell fate decisions.

Extensive analysis of another ctenophore species, *Pleurobrachia bachei*, was performed by Leonid Moroz, Andrea Kohn, Matthew Citarella, Anastasia Grigorenko, Kevin Kocot, Ken Halanych and Evgeny Rogaev (University of Florida, USA; University of Massachusetts Medical School, USA; University of Alabama, USA). These investigators sequenced major adult tissues as well as several developmental stages using 454/Roche (Branford, Conneticut, USA) and Illumina (San Diego, California, USA) technologies (~approximately 1,000× coverage) followed by RNAseq transcriptomic analysis (approximately 2000× coverage). Phylogenomic analysis of *Pleurobrachia *and 45 other organisms revealed that ctenophores occupy the basal-most position in the animal kingdom, and subsequent examination of select gene families further supported this conclusion.

Moroz *et al*. also investigated origins of the nervous systems of ctenophores. They have found that the ctenophore neural system is much more complex than that of sponges, and that many of the neural molecules present in ctenophores are novel. In some cases, these neural proteins have been highly conserved. For example, L-glutamate and secretory peptides were similar to those of bilaterians and may have been among the earliest to evolve in animals. Finally, Moroz *et al*. have hypothesized that the ctenophore nervous system evolved independently and that both the sponges and *Trichoplax *likely experienced extensive loss of neural-like function.

In addition to ctenophores, several investigators examined cnidarian development. A functional analysis of the sea anemone *Nematostella vectensis *by Mark Martindale, Tim Dubuc and Dave Simmons (University of Hawaii, USA) examined roles of the Hox genes during axial patterning. This group focused on two genes: *Ax1*, an aborally expressed "posterior" Hox gene, and *Ax6*, an orally expressed "anterior" Hox gene. As anticipated, knock-down of Ax1 affected aboral structures. The apical tuft failed to form and settlement/metamorphosis was disrupted. In addition, embryos exhibited increased expression of oral markers and the duration of gastrulation was extended. Alternately, overexpressing Ax1 resulted in down-regulation of oral markers and inhibited Ax6 expression. When Martindale *et al*. overexpressed the "anterior" Hox gene Ax6 aboral markers were down-regulated. In these embryos, the pharyngeal region was expanded, oral markers were up-regulated, and misexpression of Ax6 seemed to activate expression of Ax1. Based on the findings that overexpression/inhibition of each Hox gene affected the expected domains and gene targets and that misexpression of one Hox homologue affected expression of the other, Martindale *et al*. concluded that a simple "Hox code," possibly antecedent to that of bilaterians, exists in *Nematostella*.

## Making headway in the Lophotrochozoa

Given the prior paucity of attention to lophotrochozoan development, it was exciting to see new advances in this area. In a particularly insightful study, Aldine Amiel, Jonathan Henry and Elaine Seaver (University of Hawaii, USA) identified the organizer in the polychaete, *Capitella teleta*. As in other spiralians, organizer activity was derived from D quadrant cells. But unlike molluscs, organizer activity in *Capitella *arose, not in 3D or 4d cells, but, earlier in development, from the 2d cell. In a related talk, Emi Yamaguchi and Elaine Seaver (University of Hawaii, USA) also examined eye formation in *C. teleta*. Although the cells specifying eyes in molluscs and *Capitella *are identical (1a and 1c specify the left and right eye respectively), they convincingly demonstrated that cell 1b in *Capitella *regulates compensation for loss of either 1a or 1c. In similar experiments, the mollusc *Ilyanassa *failed to regulate for loss of the eye-generating cell. These findings are compelling, and it will be exciting to learn how mechanisms of cell fate specification have evolved in these spiralian lines.

One of the most exciting talks in the Cell Differentiation session examined reproductive development of the simultaneous hermaphrodite *Schmidtea mediterranea *(Tracy Chong and Phillip Newmark; University of Illinois, Urbana-Champaign, USA). This planarian expresses Smed-dm4, a transcription factor that regulates sexual determination and/or differentiation across different phyla. In *Schmidtea*, Smed-dm4 mRNA was detected both in the male reproductive structures (testes, seminal vesicles, penis papilla, sperm ducts) and in a subset of neurons present in the brain. To test whether Smed-dm4 regulated determination and/or differentiation of both male and female reproductive systems, RNAi knock-down was performed in hatchlings and sexually immature planarians. Early loss of Smed-dm4 (hatchlings) disrupted establishment of both male and female reproductive systems, but later loss only prevented differentiation of the testes, leading Chong and Newmark to conclude that sex-specific pathways exist in this simultaneous hermaphrodite. They have shown that Smed-dm4 initially affects establishment of both the male and female reproductive systems, but once these have been specified, it only influences differentiation of the male reproductive structures.

It was also fascinating to learn about progress in studies of animal regeneration. Eduardo Zattara and Alexa Bely (University of Maryland, College Park, USA) presented stunning visual footage of cell migration during regeneration in naidid oligochaetes, confirming Randolph's "migrating stem cell model," a 120-year-old idea that neoblasts (embryonic cells set aside to make ventral mesoderm) migrate along the ventral nerve cord to wound sites during regeneration where they proliferate to form the new tissue. Using pufferfish toxin, Zattara and Bely immobilized the oligochaete *Pristina leidyi *and performed 4D imaging, providing the first direct "real-time" evidence of neoblast migration during annelid regeneration. They also found that other cell types follow diverse migration routes, some moving at unusually high speeds (up to 5.2 μm/min). In a related talk, Duygu Ozpolat, Zattara and Bely (University of Maryland, College Park, USA), tested whether stem cell markers would label migrating cells in regenerating *P. leidyi*. Individual cells appearing to move between the gut and ventral nerve cord expressed *piwi *as did blastema cells, but other migrating cells lacked *piwi, nanos *and *vasa *expression, suggesting that at least two distinct cell types respond during regeneration.

Other talks addressed brachiopod and phoronid development. Yale Passamaneck, Sabrina Schiemann, Mark Martindale and Andreas Hejnol (University of Hawaii, USA; Sars International Centre for Marine Molecular Biology, Norway) demonstrated the importance of Notch/Delta signaling in *Terebratalia transversa *chaetal differentiation. In light of the role that this pathway plays in annelid chaetal formation, these results are particularly interesting, providing further evidence to evaluate whether chaetae in annelids and brachiopods are convergent structures or true homologues. Also, Scott Santagata (Long Island University, USA) compared cleavage patterns of brachiopods and phoronids. Using time-lapse video to examine spiral cleavage in *Phoronis pallida*, he concluded that onset of spiral cleavage is not obvious until the fourth cleavage and that earlier divisions resemble radial cleavage. In reviewing patterns reported in other phoronids and brachiopods, he found that radial-like and spiral-like cell divisions could occur within a single species as well as among species. He has hypothesized that the occurrence of both patterns in the same species may be a heritable characteristic (similar to production of dextral versus sinistral forms of the snail *Lymnaea)*. Based on this work, he also hypothesized that phoronids and brachiopods evolved from an ancestor that exhibited a form of spiral cleavage.

Finally, Andrea Kohn's investigation of molluscan nervous systems reiterated what a truly diverse group this is. In collaboration with Kevin Kocot, Ken Halanych, Matthew Citarella, Rhanor Gillette, Jonathan Sweedler and Leonid Moroz (University of Florida, USA; University of Illinois, Urbana-Champaign, USA) she characterized signaling peptides present in molluscan genomes. In this massive study, deep transcriptomic sequencing was performed on more than 40 molluscs. After identifying candidates, investigators performed *in situ *hybridizations to verify neuron-specific expression, identified neuropeptide-like molecules differentially expressed during development, and used mass spectrometry to confirm expression of peptides of interest. Ultimately, more than 5 million ESTs from 12 gastropod and cephalopod species were compared to sequences in *Aplysia *to identify conserved homologues. They have concluded that: A) the most conserved neuropeptides act in buccal feeding, movement of the foot, and release of mucus; B) pain and memory circuits appear to be fast-evolving and not well-conserved; C) molecular complexity of memory forming circuits in *Aplysia *is quite high with over 70 messengers acting at these synapses; and D) phylogenomic analysis suggests that the complex brain of molluscs evolved independently four to five times.

## Exploring the Ecdysozoa

In a study examining evolution of vulval development in nematodes, David Matus, Mary Yang, Emily Chang and David Sherwood (Duke University, USA) compared processes necessary for uterine-vulval contact in 17 rhabditid species. During larval development, the anchor cell (AC), a cell found in the somatic gonad, induces epidermal cells to form the vulva. The AC then extends through two basement membranes to invade the epidermal layer, initiating direct contact between the gonadal and epithelial regions. Although rhabditid nematodes diverged over hundreds of millions of years and are known to differ in vulval morphology, Matus *et al*. found that anchor cell invasion was surprisingly similar in all species examined. Based on this, Matus *et al*. have concluded that anchor cell invasion and stabilization of the sliding basement membranes after invasion is under strong selective pressure to be maintained.

## Invertebrate deuterostomes: tales of sea squirt tails and sea urchin cells

Insights into the evolution of the Deuterostomata continue to arise with analysis of genomic and transcriptomic data. This year, a number of the presentations/posters examined the evolution of tail loss in ascidians. Tail loss has occurred in ascidian tadpole larvae at least four times. Although most of the 3,000+ species have larvae with tails, 15 species have been described to produce tailless larvae and most of these are molgulids. C. Titus Brown, Elijah Lowe, Kanchan Pavangadkar, Max Maliska and Billie Swalla (Michigan State University/BEACON Center, USA; University of Washington, USA) compared transcriptomes of the ascideans *Molgula oculata *(tailed) and *M. occulta *(tailless). Because no reference transcriptome existed for these species, *de novo *assembly of transcripts was necessary. Although costs to sequence the transcriptome have recently dropped, expenses associated with data analysis remain high. Brown *et al*. developed an approach allowing researchers to obtain allelotypes without a reference genome. Using digital normalization to discard high coverage reads, this group was able to reduce their dataset to a manageable size, greatly reducing the cost of RNAseq analysis and allowing them to identify mutations associated with ascidian tail loss. Preliminary results indicated that most genes expressed in the larvae were maternally derived and that both tailed and tailless larvae expressed similar notochord pathway genes. In a second study associated with this project, Maliska and Swalla (University of Washington, USA) used the transcriptomic data to compare expression of genes associated with metamorphosis in *M. oculata/occulta *hybrids and *M. oculata *(gastrula, neurula and tailbud stages). They found that META-2, mannose binding lectin (MBL) and echinonectin were expressed earlier in tailed and tailless molgulids than in other ascidians, and hypothesized that early activation of metamorphosis is linked to evolution of tailless larvae in molgulids.

Other investigations focused on the intricate interactions regulating gene transcription during development. Joel Smith (Marine Biological Laboratory, Woods Hole, USA) once again illuminated the audience with his systems analyses of marine invertebrates. In his talk, Smith described regulatory interactions occurring in the sea urchin *Strongylocentrotus purpuratus *during early embryogenesis, particularly during specification of the primary mesenchyme. By comparing pathways of the *S. purpuratus *network to those found in echinoderms that do not produce primary mesenchyme, his lab is gaining valuable insights into the evolution of gene regulatory networks. In addition, he stressed the value of *Nematostella vectensis *as a model for investigating the evolution of these networks in the Metazoa.

In other key talks, Billie Swalla (University of Washington, USA) presented an intriguing overview of chordate evolution, re-examining origins of the notochord and Brad Davidson (University of Arizona, USA) described how "paired input" aided in the temporal control of Ets1/2 transcriptional activity during early versus late heart formation in the ascidian *Ciona intestinalis*,

## Vertebrate morphology: bird beaks, bat wings and lizard legs

For those interested in the evolution of vertebrate development, the vertebrate morphogenesis sessions were well worthwhile. In a highly attended talk, Arkhat Abzhanov (Harvard University, USA) fascinated the audience with his studies of the evolution of birds. Using developmental and morphometric approaches, he explained the modular nature of pathways regulating the size and shape of beak diversity in Darwin's finches and examined how craniofacial structures specific to birds could have evolved given ancestral features present in basal archosaurs and theropod dinosaurs.

This talk was immediately followed by an intriguing study of the biomechanics/development of wings in short-tailed bats (*Carollia *sp.). Lisa Cooper, John Jast, Richard Behringer, Chris Cretekos, John Rasweiler and Karen Sears (University of Illinois, Urbana-Champaign, USA; University of Texas, USA; University of Idaho, USA; SUNY Downstate Medical Center, USA), found that the humerus, radius and metacarpals of bat wings are much more flexible than those of terrestrial rodents. Nano-identification and whole bone bending tests demonstrated that bat wing metacarpals are only 40% as stiff as those in mice and 36% as hard, making bat wings much more flexible. Cross-sectional analysis revealed that medullary cavities in wing bones are also 8 to 40% larger than those of rodents. Counter to expectations that increased cell proliferation occurs at the growth plate during bat wing development, investigators found earlier onset of diaphysial elongation and ossification of bones in bats than in rodents. Bats experience rapid osteogenesis before birth and appositional growth is decreased relative to that in mice.

Later in the week, at the Evolution of Development and Genomics session, Carlos Infante, Jonathan Losos, Douglas Menke and David Kingsley (University of Georgia, USA; Harvard University, USA; Stanford University, USA) described work in the lizard genus *Anolis *that will test whether independent, repeated evolution of similar morphologies relies on shared mechanisms or unique developmental mechanisms. Evolving independently on a number of islands in the Greater Antilles, the genus *Anolis *underwent an adaptive radiation; ultimately producing a number of "ecomorphs," species morphologically specialized to a specific habitat (for example, among twigs, along tree trunks). In this elegant study, Infante *et al*. focused on long-limbed and short-limbed ecomorphs. First, they identified sequences of forelimb and hindlimb specific transcription factors in the chick and mouse as well as similar sequences in the long-limbed and short-limbed anoles. Preliminary studies revealed that hindlimb enhancer B (HLEB), an enhancer downstream of Tbx4, differed in long- and short-limbed species. By screening over 100 *Anolis *species for HLEB and amplifying it in approximately 80 species, they showed that HLEB deletions were limited to short-limbed species (three of seven short-limbed *Anolis *clades). Infante also discussed use of chromatin immunoprecipitation followed by high-throughput sequencing (ChIP-Seq) to identify enhancers of the hindlimb-specific transcription factor Pitx1. Instead of relying on identification of conserved regions (which would overlook enhancers unique to *Anolis *or squamates), this approach allows genome-wide scans to identify targets of Pitx1 to be tested for enhancer activity, generating long lists of enhancers that may work together in the evolution of *Anolis *limb morphology.

## Summary

This report only highlights a few of the many intriguing presentations that were offered at this year's SICB Annual meeting. Clearly the availability of new gene/genome sequencing technologies continues to make a major impact in the field of evolutionary development. Also, many labs are creatively adapting classical approaches to gain fascinating new insights on an expanding number of new biological systems. As these laboratories continue to examine the evolution of developmental systems, understanding of this field and the origins of biological diversity continues to grow. It should be exciting to learn what has been gained by the 2013 meeting in San Francisco, California.

## Abbreviations

AC: anchor cell; ES: embryonic stem; HLEB: hindlimb enhancer B; *Klf: Kruppel-like factor; *MBL: mannose binding lectin.

## Competing interests

The authors declare that they have no competing interests.

## Authors' contributions

This manuscript was written by CA Byrum.

## Authors' information

College of Charleston, Rita Liddy Hollings Science Center, 58 Coming St., Room 214, Charleston, SC 29401, USA, email at byrumc@cofc.edu.

